# Evolutionary Toxicology: Population-Level Effects of Chronic Contaminant Exposure on the Marsh Frogs (*Rana ridibunda*) of Azerbaijan

**DOI:** 10.1289/ehp.8404

**Published:** 2005-12-15

**Authors:** Cole W. Matson, Megan M. Lambert, Thomas J. McDonald, Robin L. Autenrieth, Kirby C. Donnelly, Arif Islamzadeh, Dmitri I. Politov, John W. Bickham

**Affiliations:** 1 Department of Wildlife and Fisheries Sciences,; 2 School of Rural Public Health,; 3 Department of Civil Engineering and; 4 Department of Veterinary Anatomy and Public Health, Texas A&M University, College Station, Texas, USA; 5 Sumgayit Centre for Environmental Rehabilitation, Sumgayit, Azerbaijan; 6 Vavilov Institute of General Genetics, Russian Academy of Sciences, Moscow, Russia

**Keywords:** Azerbaijan, complex mixtures, evolutionary toxicology, heteroplasmy, mutation rate, Rana ridibunda

## Abstract

We used molecular methods and population genetic analyses to study the effects of chronic contaminant exposure in marsh frogs from Sumgayit, Azerbaijan. Marsh frogs inhabiting wetlands in Sumgayit are exposed to complex mixtures of chemical contaminants, including petroleum products, pesticides, heavy metals, and many other industrial chemicals. Previous results documented elevated estimates of genetic damage in marsh frogs from the two most heavily contaminated sites. Based on mitochondrial DNA (mtDNA) control region sequence data, the Sumgayit region has reduced levels of genetic diversity, likely due to environmental degradation. The Sumgayit region also acts as an ecological sink, with levels of gene flow into the region exceeding gene flow out of the region. Additionally, localized mtDNA heteroplasmy and diversity patterns suggest that one of the most severely contaminated sites in Sumgayit is acting as a source of new mutations resulting from an increased mutation rate. This study provides an integrated method for assessing the cumulative population impacts of chronic contaminant exposure by studying both population genetic and evolutionary effects.

Evolutionary toxicology is the study of the effects of contaminants on the genetics of natural populations and is rapidly becoming an essential component of ecotoxicology. The use of population genetic data to investigate effects of chronic contaminant exposure has proven effective in studies representing a diversity of organisms and ecological settings. Molecular genetics is a powerful tool to identify reductions in genetic diversity (Murdoch and Hebert 1994), contamination-induced natural selection (Bridges and Semlitsch 2001; Foré et al. 1995a, 1995b; Peles et al. 2003; Theodorakis and Shugart 1997), ecological sinks (Baker et al. 2001; Matson et al. 2000; Theodorakis et al. 2001), and increased mutation rates (Ellegren et al. 1997; Forster et al. 2002; Somers et al. 2002). The application of evolutionary or population genetic approaches to problems in ecotoxicology has been reviewed in several articles (Belfiore and Anderson 2001; Bickham et al. 2000; Bickham and Smolen 1994; Hebert and Luiker 1996; Van Straalen and Timmermans 2002). Bickham et al. (2000) provided a thorough discussion of the complex pathways through which contaminants can affect wildlife populations.

Three specific population genetic effects were of interest in this study, the first being genetic diversity. Environmental contaminants can lead to a reduction in genetic diversity resulting from strong selection for chemical tolerance or population declines. Reductions in fecundity or viability, or increased mortality as a result of contaminant exposure can lead to a reduction of the number of breeding adults. Semlitsch et al. (2000) were able to demonstrate that there was a fitness trade-off associated with chemical tolerance in gray tree frog (*Hyla versicolor*) tadpoles. Tadpole survival, in the absence of a chemical stressor, was negatively correlated with tolerance to the insecticide carbaryl. More complex scenarios involve ecosystem degradation leading to reductions in carrying capacity, thus resulting in smaller populations. Although population surveys are generally the most accurate means of estimating population size, in situations where no surveys were performed before contaminant exposures, as in Sumgayit, Azerbaijan, population genetic techniques are quite useful for investigating possible changes in effective population size (*N_e_*). Populations that have experienced a significant bottleneck are expected to suffer a reduction in population genetic diversity. The severity and duration of the bottlenecks will determine the amount of diversity loss (Bickham et al. 2000). Genetic diversity is generally measured as either “haplotype” or “nucleotide” diversity (Nei 1987). Gene or haplotype diversity looks only at the number and frequencies of haplotypes, whereas nucleotide diversity also includes a measure of haplotype sequence similarity (Nei 1987; Tajima 1983). Haplotype, as used throughout this article, is defined as a unique sequence of nucleotides for a defined region, providing a distinctive genetic pattern. The loss of population genetic diversity has been linked to environmental contaminants (Kopp et al. 1992; Krane et al. 1999; Murdoch and Hebert 1994; Street and Montagna 1996).

Our second effect of interest was mutation rate. Contaminant exposure can lead to an increased mutation rate that might offset some of the genetic diversity loss caused by bottlenecks. The haplotype patterns found in these populations can be informative in determining whether populations have gone through bottlenecks and/or have elevated mutation rates. Elevated levels of heteroplasmy, the presence of more than one mitochondrial DNA (mtDNA) variant within an individual, in contaminated populations suggest the presence of an elevated mutation rate (Forster et al. 2002). However, it is important to understand that different types and patterns of heteroplasmy suggest different sources. In several instances, the differences between the heteroplasmic haplotypes have been too large to suggest a simple gametic or somatic mutational event (Kvist et al. 2003; Magoulas and Zouros 1993). This pattern suggests that paternal leakage is the cause and not gametic or somatic mutation. Paternal leakage is the result of at least one mitochondrion from the sperm cell being incorporated into a fertilized egg, leading to the presence of both maternal and paternal mtDNA in the resulting offspring. Paternal leakage is a rare exception to the strictly maternal inheritance of mtDNA. Where paternal leakage is indicated, heteroplasmic haplotypes are likely to be common haplotypes (Magoulas and Zouros 1993), or they might be differentiated by more than a single mutational event (Kvist et al. 2003; Magoulas and Zouros 1993). In perhaps the most comprehensive examination of population heteroplasmy, paternal leakage was ruled out by studying people with known pedigrees (Forster et al. 2002). In that study, Forster et al. (2002) showed that heteroplasmy resulted from gametic mutations, and the heteroplasmic condition was subsequently passed down the maternal lineage. This condition was also shown to be linked to natural radiation exposure because populations from radioactive areas had an elevated frequency of heteroplasmy (Forster et al. 2002). These data combine to show that heteroplasmy can be an effective means for investigating mutation rates. Mutational heteroplasmy appears to be generally more common than complete mutations and thus a more sensitive means of detecting increased mutation rates in populations.

The third effect we wanted to investigate is whether or not contaminant exposure has resulted in an ecological sink. An ecological sink is an area that appears to provide suitable habitat, but animals fail to adequately survive and/or reproduce, and thus the population relies on immigration from source populations to maintain population numbers. Although this is in fact an ecological effect, it can be investigated using population genetics. Theodorakis et al. (2001) used population genetics analyses to investigate the genetic patterns of kangaroo rats (*Dipodomys merriami*) inhabiting radionuclide-contaminated ground-zero sites at the Nevada test site (nuclear testing site). Based on both genetic diversity and gene flow estimates, they concluded that the atomic blast sites act as ecological sinks. Additionally, they concluded that immigration into the blast sites masks the genotoxic effects of the radiation contamination on the resident kangaroo rat populations. Population genetic studies of bank voles (*Clethrionomys glareolus*) living near Chornobyl, led researchers to conclude that the most contaminated areas were likely acting as ecological sinks (Baker et al. 2001; Matson et al. 2000). Although the authors were unable to eliminate an increased mutation rate as being the cause of observed genetic patterns, subsequent heteroplasmy studies suggest that mutation rates are not elevated at Chornobyl (Wickliffe et al. 2002, 2003).

Azerbaijan is a former Soviet republic located on the western shores of the Caspian Sea. This small country is well known for its large oil reserves, which have been exploited for more than a century. Oil production, as well as the chemical and manufacturing plants of Sumgayit, has left the Apsheron Peninsula of Azerbaijan with a complex mixture of environmental contaminants. Environmental degradation and contamination in Azerbaijan, specifically in Sumgayit, have been explored recently (Andruchow 2003; Bickham et al. 2003; Matson et al. 2005a, 2005b; Shelton 2003; Swartz et al. 2003). Elevated levels of numerous potentially mutagenic contaminants, including polycyclic aromatic hydrocarbons (PAHs), mercury, polychlorinated biphenyls, and organochlorines, have been documented in wildlife, sediments, and soils from Sumgayit and the capitol city of Baku (Bickham et al. 1998; Matson et al. 2005a, 2005b; Swartz et al. 2003). There is no doubt that the contaminants in Sumgayit affect both wildlife and humans; however, there is still debate regarding the magnitude and types of effects. Our previous studies have shown elevated levels of chromosomal damage in wildlife (Matson et al. 2005a, 2005b), and a recent study has also shown increased cancer rates in humans (Andruchow 2003). The complex mixtures of contaminants found in Sumgayit make it difficult to prove causal links between individual contaminants and observed effects. Therefore, associations and correlations are used to identify contaminants that are likely to be involved in a particular biomarker effect. In addition, it is difficult to fully understand what the ultimate consequences of increased genetic damage as well as other effects might be on individuals or populations. In an earlier study, we were able to document significant environmental exposures and genetic damage in marsh frogs (*Rana ridibunda*) from several sites in the Sumgayit region of Azerbaijan (Matson et al. 2005b). The use of marsh frogs in the present study to investigate population genetic patterns allowed for a more informed interpretation of observed patterns. A discussion of the advantages of this species as an environmental model has been published elsewhere (Matson et al. 2005b).

The *Exxon Valdez* oil spill provided scientists with an *in situ* laboratory to study the impacts of long-term chronic PAH exposure to a variety of mollusks, fish, birds, and mammals (Downs et al. 2002; Esler et al. 2002; Peterson et al. 2003). Although the effects of acute exposures to oil have been well documented and are currently taken into account in ecological risk assessments, the effects of long-term chronic exposure to weathered oil is not well understood or adequately included in risk assessments. Peterson et al. (2003) recently concluded that chronic exposure to PAHs, even at concentrations of parts per billion, could have significant population consequences. These population-level impacts are the result of sublethal doses of PAHs that compromised health, growth, and reproduction. Additionally, indirect ecosystem changes contributed to population declines. These data provide strong support for including probable population-level impacts from chronic exposure to contaminants into both wildlife and human risk assessment models.

In this study, we tested three hypotheses regarding marsh frog populations in Azerbaijan. First, we wanted to determine if Sumgayit populations exhibit reduced levels of genetic diversity that could be the result of a population bottleneck. Second, we tested whether the most contaminated areas within Sumgayit are acting as sources of new mutations. Third, we investigated patterns of gene flow to determine if the Sumgayit region is acting as an ecological sink.

## Materials and Methods

### Sample collection.

**Figure 1:**
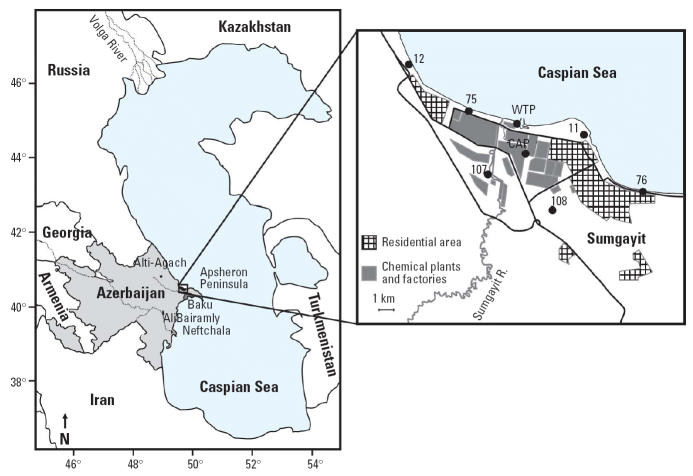
Map of Azerbaijan showing both experimental (inset: Sumgayit) and reference collection localities.

Marsh frogs were collected from a total of 11 sites between 1999 and 2002. Collecting areas consisted of marshes, ponds, and in one case a drainage ditch. A map of collecting locations is presented in Figure 1. Site coordinates are as follows: Neftchala (NEF), N 39°24′, E 49°15′; Ali Bairamly (ALI), N 39°56.777′, E 48°55.060′; Alti-Agach, N 40°53.012′, E 48°59.800′; waste-water treatment plant (WTP), N 40°36.857′, E 49°37.280′; site 11, N 40°36.380′, E 49°40.477′; site 12, N 40°38.671′, E 49°32.381′; site 75, N 40°37.402′, E 49°34.696′; site 76, N 40°34.468′, E 49°43.129′; chloralkali plant (CAP), N 40°35.788′, E 49°37.700′; site 107, N 40°35.180′, E 49°36.110′; site 108, N 40°33.917′, E 49°38.778′. Eight of the sites are located within the city of Sumgayit, and the remaining three are from reference areas. Two of the reference sites are to the south of Sumgayit (ALI and NEF); the third is to the northwest in the Caucus Mountains (Alti-Agach). Sumgayit samples were considered contaminated based on environmental chemistry data presented previously (Matson et al. 2005b) and their proximity to a large industrial area with a documented history of widespread chemical contamination (Bickham et al. 2003). Sample sizes are presented in Table 1.

Frogs were anesthetized with MS222 (CAS 886-86-2; Sigma-Aldrich Corp., St. Louis, MO, USA). Blood samples for DNA analyses were then obtained via abdominal venipuncture and placed in lysis buffer (Longmire et al. 1997). All animals included in this study were treated humanely, and every effort was made to avoid needless suffering. Specific collection and handling procedures were reviewed and approved by the Texas A&M University Laboratory Animal Care Committee. Samples were immediately refrigerated at 4°C until they were transported back to Texas A&M University for processing. Animals were then sacrificed via pithing. Frozen tissue samples were also collected and subsequently archived at Texas A&M University.

### DNA extraction and sequencing.

Total genomic DNA was extracted from blood samples using Qiagen DNeasy tissue kits (Qiagen Inc., Valencia, CA, USA) following the manufacturer’s protocol. A 788-bp fragment of mtDNA was then directly amplified using polymerase chain reaction (PCR) using primers designed specifically for *Rana ridibunda*: RANA CR-3F (5′-GAAGACCCCTTTATCACCATCG-3′) and RANA CR-4R (5′-GGGTACGATAGGGCTTATGAAT-3′). These primers amplify a region of mtDNA that includes a 63-bp portion of the 3′ end of the cytochrome *b* (*CYTB*) gene and the first 725 bp of the 5′ end of the control region. This portion of the mtDNA control region is generally referred to as the hypervariable region I (HVR1). Amplifications were performed with AmpliTaq Gold PCR Master Mix (Applied Biosystems, Foster City, CA, USA) following the manufacturer’s recommendations and a 50°C annealing temperature. Fragments were then purified using Qiagen QIAquick PCR purification kits (Qiagen Inc.).

Sequencing reactions were performed using the primer RANA CR-5F (5′-CCTTTATCACCATCGGTCAAATCG-3′) and ABI PRISM BigDye Terminator v1.1 Cycle Sequencing Kits (Applied Biosystems) following manufacturer’s recommendations. A subset of individuals was additionally sequenced using the primer RANA CR-2R (5′-GGCTTATGAATATTGCGTCGAG-3′). Unincorporated dye terminators were removed from sequencing products with Qiagen DyeEx 2.0 spin kits (Qiagen Inc.). Purified sequencing products were then analyzed on an ABI PRISM 377 DNA sequencer (Applied Biosystems). Sequence chromatographs were proofed and aligned using Sequencher software (version 4.1; Gene Codes Corp., Ann Arbor, MI, USA).

In addition to our normal confirmation efforts, all ambiguous or heteroplasmic nucleotides and novel haplotypes were confirmed by sequencing in the reverse direction using RANA CR-2R. Detection limits for heteroplasmy were approximately 15–20%. Individuals with lower frequencies of secondary haplotypes were likely overlooked. Genomic DNA also was extracted from liver samples for all heteroplasmic individuals. DNA extraction, PCR, and sequencing methods were performed as with blood samples. We compared the ratio of heteroplasmic nucleotide proportions for both liver and blood samples to confirm that observed mtDNA heteroplasmy was not the result of a nuclear pseudo-gene. Because the mtDNA content of cells varies between tissues depending on metabolic activity, using different tissues for source DNA yields different ratios of nuclear to mitochondrial DNA. As a result, if our heteroplasmy was the result of a pseudogene, the proportions of heteroplasmic nucleotides would vary from tissue to tissue depending on the ratio of nuclear and mitochondrial DNA. Because the proportion of our heteroplasmic nucleotides did not vary between blood and liver samples, which have widely different ratios of nuclear and mitochondrial DNA, we concluded that observed heteroplasmy was not the result of a nuclear pseudogene.

The final mtDNA fragment we used for analyses was 582 bp and included the terminal 32 bp of *CYTB* and the adjacent 550 bp of the control region. There are no tRNA genes separating *CYTB* from the control region in many ranid species, including *R. ridibunda* (Sumida et al. 2000, 2001). The nucleotide sequences of each haplotype were deposited in GenBank (accession numbers AY554011–AY554027; GenBank, National Center for Biotechnology Information, Bethesda, MD, USA).

### Data analysis.

We analyzed sequence data for both population and regional groupings. Population data were analyzed only for sites with at least 10 individuals. The two cases in which there was not an adequate population sample size for analysis (sites 107 and 108), the individuals were used only in the regional analyses. Haplotype diversity (*h*) and nucleotide diversity (π) were calculated at both scales using ARLEQUIN (version 2.000) (Schneider et al. 2000). We also used ARLEQUIN to calculate linearized F_ST_ values and migration estimates (Slatkin 1991, 1995). MIGRATE (version 1.7.3; Beerli 2003) was used to estimate *N_e_* and number of effective migrants (*Nm*) for regional groupings using a maximum likelihood methodology (Beerli and Felsenstein 2001). A minimum spanning network was constructed manually with all equally parsimonious connections being represented.

## Results

**Figure 2:**
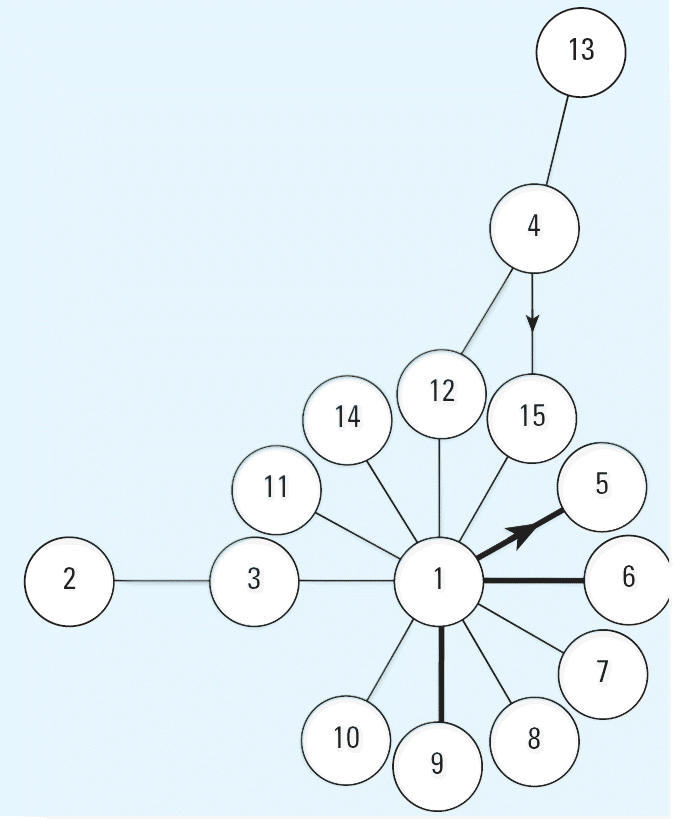
Minimum spanning network of all *R. ridi-bunda* haplotypes from Azerbaijan. Each connection represents a single mutational event, with thick lines representing transversions. Arrowheads on connections represent heteroplasmic conditions between the two haplotypes and indicate the inferred mutational direction.

A total of 15 haplotypes were documented in *R. ridibunda* populations from Azerbaijan. Both population and regional haplotype counts are presented in Table 1. There were 13 variable nucleotide positions identified. Of the variable nucleotide positions, 10 were transitions and three were transversions. One of the transitions was located within *CYTB*, whereas the remaining variable positions were located in the control region. In addition to the frogs with one of the 15 haplotypes, six frogs were found to be heteroplasmic for haplotypes 1 and 5, and two frogs were found to be heteroplasmic for haplotypes 4 and 15. Secondary heteroplasmic haplotype proportions ranged from approximately 15 to 45%. A minimum spanning network of all *R. ridibunda* haplotypes from Azerbaijan is presented in Figure 2.

**Figure 3:**
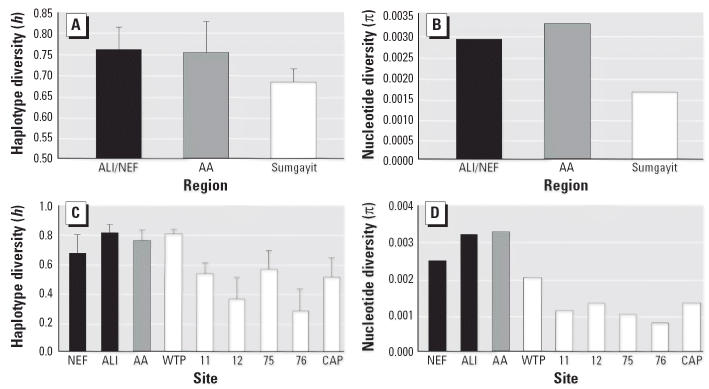
Haplotype (*A*) and nucleotide (*B*) diversity for reference (shaded) and experimental (white) regions and haplotype (*C*) and nucleotide (*D*) diversity for reference and experimental sites. Error bars represent SE.

Regional and population diversity estimates are presented in Table 1. At a regional scale, Sumgayit had reduced levels of both haplotype and nucleotide diversity relative to both reference regions (Figure 3). At the population level, five out of the six sites in Sumgayit had lower haplotype diversity than did our three reference populations (Figure 3). The WTP site was the only Sumgayit population that appeared to have relatively normal haplotype diversity. However, all of the Sumgayit populations showed reduced levels of nucleotide diversity relative to the reference populations (Figure 3).

**Figure 4:**
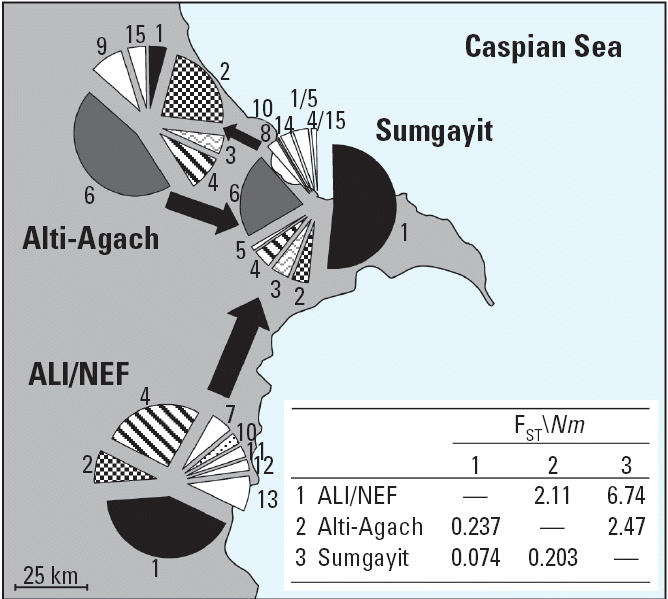
Regional haplotype patterns and estimates of genetic differentiation (F_ST_) and gene flow (*Nm*). Shared haplotypes are shaded or patterned; unique haplotypes are white.

To investigate population substructure at a regional scale, F_ST_ (a measure of population genetic divergence) estimates were computed and are presented in Figure 4. Regional estimates of *Nm* were calculated using two different methods: a traditional method using F_ST_ values (Slatkin 1991) and a maximum likelihood (ML) method (Beerli and Felsenstein 2001). Traditional estimates of *Nm* only estimate total gene flow between populations (Figure 4), whereas the ML method computes directional estimates of gene flow between populations (Table 2). Both methods result in the highest estimates of gene flow occurring between Sumgayit and the ALI/NEF region, and the lowest estimates occurred between ALI/NEF and Alti-Agach. ML estimates suggest that gene flow between Sumgayit and ALI/NEF is almost entirely movement from ALI/NEF into Sumgayit, and that gene flow between Sumgayit and Alti-Agach is weighted toward emigration out of Sumgayit. The directional nature of gene flow from traditional estimates must be deduced from population haplotype frequencies and patterns. Our interpretation of haplotype patterns suggests that gene flow among the regions is directional with the majority resulting from immigration into Sumgayit. Haplotypes that are common in the north are also common in Sumgayit but do not penetrate to the south. Haplotypes that are common in the south are also found in Sumgayit but do not penetrate to the north. Therefore, it appears that gene flow (migration) is into Sumgayit from both directions, but not out of Sumgayit in either direction, or at least at a much lower rate (Figure 4).

Effective population size (*N_e_*) was estimated for each region using an ML approach. Theta (θ), in the case of mtDNA, is equal to *N_e_* multiplied by the mutation rate (μ). Mutation rates are generally assumed to be equal among populations. Therefore, we used estimates of θ to compare *N_e_* (Table 2). These estimates suggest that the ALI/NEF region has the largest population size, followed by Alti-Agach and Sumgayit, respectively.

## Discussion

Patterns of regional and population diversity support the hypothesis that marsh frog populations from Sumgayit have reduced genetic diversity, likely through population reductions and/or bottlenecks. The Sumgayit region has reduced levels of both haplotype and nucleotide diversity relative to both ALI/NEF and Alti-Agach (Figure 3). Additionally, all of the experimental sites within Sumgayit exhibit reduced levels of nucleotide diversity relative to reference sites. The WTP is the only Sumgayit population to not also show reduced haplotype diversity. Although selection and/or other factors cannot be totally excluded, the observed reduction in diversity is likely the result of genetic drift exacerbated by declines in *N_e_*. Although selective pressures can be high in contaminated environments, complex mixtures of contaminants and constantly changing environmental conditions are less likely to result in a loss of genetic diversity via selection than through genetic drift acting on a small population. We conclude that the observed loss of diversity is likely the result of population declines, and that environmental degradation (Bickham et al. 2003; Matson et al. 2005a, 2005b; Swartz et al. 2003) is the most likely cause of the regional reductions of genetic diversity.

Patterns of diversity and gene flow can be used to evaluate whether an area is acting as an ecological sink. Reduced genetic diversity is not necessary to identify an area as an ecological sink. In fact, a sink could result in increased diversity of a local population if immigration is from multiple populations. Population history, including whether the population is recovering or declining, and genetic patterns are important factors when interpreting population diversity levels. A recovering population that has lost diversity during previous bottlenecks would be expected to continue showing reduced genetic diversity unless new diversity was being introduced to the population through gene flow or new mutations. If a population were extirpated and subsequently recolonized from a single adjacent population, one would expect to see reduced levels of genetic diversity. This process would be expected to yield very high estimates of gene flow and low genetic differentiation among populations. If the extirpated population were recolonized from multiple adjacent populations, one might expect to have normal or elevated levels of genetic diversity relative to reference populations. Recent population genetic investigations of possible ecological sinks have concluded that observed genetic patterns suggested population extirpation and a subsequent recolonization from adjacent populations (Baker et al. 2001; Matson et al. 2000; Theodorakis et al. 2001). The genetic patterns of the Sumgayit population do not support the hypothesis that frogs were extirpated from the area and subsequently recolonized from one or more surrounding populations. This scenario would be expected to result in a current population with relatively normal or elevated levels of both haplotype and nucleotide diversity. Additionally, under the extirpation hypothesis one would not expect to find multiple unique haplotypes in Sumgayit.

Patterns of haplotype distributions, gene flow, and diversity indices are consistent with the Sumgayit region being an ecological sink. Population genetic patterns suggest that most gene flow occurring between Sumgayit and ALI/NEF is actually immigration into Sumgayit (Table 2, Figure 4). This regional comparison is probably the most significant in that these regions are ecologically similar lowland marsh habitats that have the highest levels of gene flow among regions. Although both of our methods for estimating gene flow suggest that migration between Sumgayit and Alti-Agach is less than that between Sumgayit and ALI/NEF, directional gene flow estimates between Sumgayit and Alti-Agach are contradictory. However, based on the distribution of common haplotypes, we conclude that migration is primarily from Alti-Agach to Sumgayit. Regional genetic diversity estimates support our hypothesis that diversity was lost in Sumgayit because of reductions in *N_e_* and that the population was almost certainly not extirpated. We have shown that many environmental contaminant concentrations have been reduced significantly in the last decade (Matson et al. 2005a), and Bickham et al. (2003) discussed the history of Sumgayit, including the economic decline and subsequent closings and reduced production at most plants and factories after the collapse of the Soviet Union. This reduction in new pollution and the transport and weathering of existing contaminants has allowed for improvements in environmental quality in Sumgayit. This improvement in environmental health has likely allowed marsh frog populations to begin to recover from decades of terrible environmental conditions. Although conditions are improving, biomarkers of genetic damage still suggest that frogs in Sumgayit are affected by contaminant exposure. Even with the environmental improvements in Sumgayit, population genetic patterns support the hypothesis that Sumgayit is acting as an ecological sink.

Although regional data suggest that frog populations in Sumgayit are being affected by long-term contaminant exposure, we wanted to investigate these effects at individual sites throughout the region. As described above, genetic diversity estimates revealed reduced levels of both haplotype and nucleotide diversity at every site within the Sumgayit region except the WTP. The WTP was shown to have reduced levels of nucleotide diversity but normal levels of haplotype diversity. In a population with reduced haplotype and nucleotide diversity, an elevated mutation rate would be expected to generate an increase in haplotypic diversity more rapidly than an increase in nucleotide diversity. We propose that the WTP is actually a source of new mutations in the Sumgayit region. Based on patterns of haplotype and nucleotide diversity, this argument would not be compelling. However, we were able to discover significant evidence to support this hypothesis. Eight individuals with mtDNA heteroplasmy were observed in this study. All of the heteroplasmic individuals were from the Sumgayit region, and seven of the eight were found at the WTP.

Heteroplasmy can be caused by a few different mechanisms. The two most probable mechanisms are paternal leakage and new mutations. It is not always possible to determine which mechanism led to any observed heteroplasmy. Paternal leakage was concluded to be the most likely cause of observed hetero-plasmy in two studies (Kvist et al. 2003; Magoulas and Zouros 1993). Magoulas and Zouros (1993) justified their conclusion that paternal leakage was the cause of observed heteroplasmy in anchovy (*Engraulis encrasicolus*) because the haplotypes involved were all common haplotypes in the population and were phylogenetically separated by several mutational events. Kvist et al. (2003) concluded that heteroplasmy observed in populations of great tit (*Parus major*) was the result of paternal leakage based on the phylogenetic relationship of the heteroplasmic haplotypes. In this case, the haplotypes were representative of two different subspecies and the heteroplasmic individuals were concluded to be hybrids. In our heteroplasmic marsh frogs, the haplotypes vary by only a single nucleotide in both the 1/5 and 4/15 forms (Figure 2). Additionally, the 4/15 heteroplasmy is only found at the WTP, and haplotype 15 has not been documented within the Sumgayit region. The 1/5 heteroplasmy is slightly more complicated in that both haplotypes are present at the WTP, although haplotype 5 is only present at a low frequency. The most convincing evidence that the observed heteroplasmy from the WTP is the result of new mutations resulting from contaminant exposure is that seven out of the eight heteroplasmic individuals were found at the WTP (Table 1). The remaining heteroplasmic frog was collected at the closest site to the west (site 75). Our data suggest that there is a significant amount of gene flow between these two populations. Therefore, we cannot rule out the possibility that the single heteroplasmic frog collected away from the WTP could have actually originated from or is a descendant of a female from the WTP. If paternal leakage was the mechanism by which heteroplasmy arose, one would expect to see this phenomenon in all populations at a comparable rate of occurrence. In fact, the observed frequencies of heteroplasmic frogs in Azerbaijan populations are significantly different from expected values under a random distribution model (χ^2^, *p* = 0.031). We cannot rule out the possibility that contaminants at the WTP have somehow interfered with the normal processes that prevent paternal transmission of mtDNA. However, to our knowledge no study has yet shown a relationship between contaminant exposure and the disruption of strict maternal inheritance of mtDNA. Thus, we conclude that paternal leakage is likely not the cause of the observed heteroplasmy in marsh frogs from Sumgayit. We hypothesize that observed heteroplasmy is the transitional state resulting from new mtDNA mutations. It is impossible to predict whether these heteroplasmic mutations will become full mutations or if they will be lost in future generations. Forster et al. (2002) showed that mutational heteroplasmy can continue through multiple generations in humans without reaching fixation. In fact, they found 23 heteroplasmic mutations but failed to find a single full mutation.

We were unable to determine the number of mutational events that led to these observed heteroplasmic frogs. We hypothesize that all of the individuals sharing a similar heteroplasmic condition are the result of single mutational events. We know that at least two independent events would be required to produce the two observed heteroplasmic forms. At least one other mutational event is required to account for the individual from site 75 if this frog is not the result of gene flow with the WTP. We believe the likelihood of migration to be much higher than another independent identical mutational event.

An increase in the frequency of heteroplasmy is sufficient to suggest that Sumgayit is acting as a source of new mutations. Evidence of genetic damage and even genomic instability in frogs from populations within Sumgayit indicates that the increased mutation rate at Sumgayit is likely an impact of contaminant exposure (Matson et al. 2005b). Although PAHs and mercury have been linked to the elevated genetic damage in marsh frogs from Sumgayit (Matson et al. 2005b), the complex mixture of contaminants found at the WTP makes it impossible to establish a causal relationship with any particular individual or group of contaminants and the elevated mutation rate.

In addition to the elevated contaminant concentrations found in Sumgayit, populations of marsh frogs in Sumgayit reveal the population genetic and evolutionary impacts of living in a contaminated environment in several ways. Genetic damage is elevated in both the WTP and CAP in Sumgayit (Matson et al. 2005b). The WTP seems to be a source of new mutations as a result of an increased mutation rate. The Sumgayit region has reduced levels of genetic diversity that is detrimental to a population’s potential to adapt and survive. Finally, the Sumgayit region seems to act as an ecological sink. The effects we have documented at multiple levels of biologic organization combine to provide real insight into the effects of long-term contaminant exposure on wildlife populations.

## Figures and Tables

**Table 1:** Population and regional haplotype counts, sample sizes (*n*), and haplotype (*h*) and nucleotide (π) diversity estimates (SE).

	Haplotype																		Diversity indices	
Population	1	2	3	4	5	6	7	8	9	10	11	12	13	14	15	4/15	1/5	n	*h* (SE)	π(SE)
NEF	6	1	—	3	—	—	1	—	—	—	—	—	—	—	—	—	—	11	0.673 (0.123)	0.00245 (0.00182)
ALI	8	2	—	6	—	—	1	—	—	1	1	1	3	—	—	—	—	23	0.814 (0.055)	0.00318 (0.00211)
AA	1	5	1	2	—	10	—	—	2	—	—	—	—	—	1	—	—	22	0.753 (0.076)	0.00327 (0.00216)
WTP	17	3	2	3	2	15	—	1	—	—	—	—	—	2	—	2	5	52	0.803 (0.036)	0.00202 (0.00147)
Site 11	7	—	2	—	—	15	—	—	—	—	—	—	—	—	—	—	—	24	0.540 (0.082)	0.00112 (0.00100)
Site 12	12	2	—	1	—	—	—	—	—	—	—	—	—	—	—	—	—	15	0.362 (0.145)	0.00132 (0.00114)
Site 75	13	—	—	—	—	1	—	3	—	—	—	—	—	2	—	—	1	20	0.568 (0.119)	0.00096 (0.00092)
Site 76	12	—	1	1	—	—	—	—	—	—	—	—	—	—	—	—	—	14	0.275 (0.148)	0.00074 (0.00079)
CAP	14	1	2	1	—	2	—	—	—	—	—	—	—	—	—	—	—	20	0.511 (0.128)	0.00131 (0.00112)
Site 107	2	1	—	1	—	1	—	—	—	—	—	—	—	—	—	—	—	5	—	—
Site 108	—	—	—	—	—	—	—	—	—	1	—	—	—	—	—	—	—	1	—	—
Region																				
NEF/ALI	14	3	—	9	—	—	2	—	—	1	1	1	3	—	—	—	—	34	0.761 (0.053)	0.00292 (0.00194)
AA	1	5	1	2	—	10	—	—	2	—	—	—	—	—	1	—	—	22	0.753 (0.076)	0.00327 (0.00216)
Sumgayit	77	7	7	7	2	34	—	4	—	1	—	—	—	4	—	2	6	151	0.684 (0.033)	0.00164 (0.00125)
Total	92	15	8	18	2	44	2	4	2	2	1	1	3	4	1	2	6	207	0.744 (0.025)	0.00216 (0.00151)

Abbreviations: AA, Alti-Agach; —, not detected.

**Table 2:** ML estimates of θ (where θ = *N_e_*μ) and estimates of *Nm* for marsh frog populations in Azerbaijan.

			*Nm*		
	Population (receiving)	θ (*N_e_*μ)	1	2	3
1	ALI/NEF	0.00125	—	0.16	0.00
2	Alti-Agach	0.00107	0.00	—	2.88
3	Sumgayit	0.00071	5.05	0.46	—

Ln(likelihood) = 55.798.
